# Comparative Study of sEMG Feature Evaluation Methods Based on the Hand Gesture Classification Performance

**DOI:** 10.3390/s24113638

**Published:** 2024-06-04

**Authors:** Hiba Hellara, Rim Barioul, Salwa Sahnoun, Ahmed Fakhfakh, Olfa Kanoun

**Affiliations:** 1 Professorship for Measurements and Sensor Technology, Chemnitz University of Technology, Rechenhainer Straße 70, 09126 Chemnitz, Germany; hiba.hellara@etit.tu-chemnitz.de (H.H.); rim.barioul@ieee.org (R.B.); 2 Laboratory of Signals, Systems, Artificial Intelligence and Networks, Digital Research Centre of Sfax, National School of Electronics and Telecommunications of Sfax, University of Sfax, Technopole of Sfax, Sfax 3021, Tunisia; salwa.sahnoun@enetcom.usf.tn (S.S.); ahmed.fakhfakh@enetcom.usf.tn (A.F.)

**Keywords:** myography, feature extraction, surface electromyography, feature evaluation, feature selection, gesture recognition

## Abstract

Effective feature extraction and selection are crucial for the accurate classification and prediction of hand gestures based on electromyographic signals. In this paper, we systematically compare six filter and wrapper feature evaluation methods and investigate their respective impacts on the accuracy of gesture recognition. The investigation is based on several benchmark datasets and one real hand gesture dataset, including 15 hand force exercises collected from 14 healthy subjects using eight commercial sEMG sensors. A total of 37 time- and frequency-domain features were extracted from each sEMG channel. The benchmark dataset revealed that the minimum Redundancy Maximum Relevance (mRMR) feature evaluation method had the poorest performance, resulting in a decrease in classification accuracy. However, the RFE method demonstrated the potential to enhance classification accuracy across most of the datasets. It selected a feature subset comprising 65 features, which led to an accuracy of 97.14%. The Mutual Information (MI) method selected 200 features to reach an accuracy of 97.38%. The Feature Importance (FI) method reached a higher accuracy of 97.62% but selected 140 features. Further investigations have shown that selecting 65 and 75 features with the RFE methods led to an identical accuracy of 97.14%. A thorough examination of the selected features revealed the potential for three additional features from three specific sensors to enhance the classification accuracy to 97.38%. These results highlight the significance of employing an appropriate feature selection method to significantly reduce the number of necessary features while maintaining classification accuracy. They also underscore the necessity for further analysis and refinement to achieve optimal solutions.

## 1. Introduction

Hand gesture recognition based on biosignals has recently become increasingly important for its potential to significantly improve the recovery and functionality of individuals with hand-related impairments [1,2]. Hand gestures are essential to human communication and interaction, and losing or impairing them can significantly impact the quality of life of individuals [1,2,3]. Surface electromyography (sEMG) signals have found widespread applications in various domains. In rehabilitation, sEMG is utilized for biofeedback, neuromuscular disorder diagnosis, and monitoring recovery progress, particularly in conditions like stroke, spinal cord injuries, and musculoskeletal disorders [4,5]. sEMG plays a crucial role in prosthetic control, enabling the recognition of human motion intentions and facilitating the operation of prosthetic limbs, exoskeletons, and assistive devices [4,6]. Additionally, sEMG signals are employed in assistive robotics, allowing for seamless human–robot interaction and control through the interpretation of muscle activation patterns [4,6]. Furthermore, sEMG has applications in human–computer interaction systems, such as virtual reality, sign language recognition, and gaming, demonstrating its versatility across diverse fields [7]. sEMG signals offer several advantages for the classification of hand gestures, human–machine interaction, and muscle-related applications. sEMG directly measures the electrical activity produced by skeletal muscles, providing a direct interface to the neuromuscular system and the intended movements [8,9]. This allows for sEMG-based systems to capture subtle finger and hand gestures that may be difficult to detect with vision-based approaches alone. Additionally, sEMG is not susceptible to external lighting conditions or occlusions, rendering it a robust choice for use in a multitude of environments [9]. The substantial information content present in sEMG signals enables the accurate classification of a vast array of hand gestures, including individual finger movements, which is of significant value for applications, such as prosthetic hand control and human–computer interaction [8,10]. Moreover, sEMG can provide insights into muscle activation patterns, force estimation, and fatigue monitoring, rendering it a valuable tool for biomechanics research, rehabilitation, and ergonomic assessments [10]. Moreover, the EMG signal appears before the muscle contraction mechanically [11], providing information about the co-contraction level of antagonist muscles that kinematic or dynamic measures cannot calculate [12]. This information is derived from the cortically originated spectral properties of the sEMG signals, which are altered in neurological patients [13].

sEMG feature extraction is essential in hand gesture classification applications that employ sEMG signals. This process significantly enhances classification accuracy, and its importance can be viewed through various lenses. The practicality of sEMG-based hand gesture recognition is constrained by a number of limitations that need to be addressed in order to enhance its effectiveness. A primary obstacle involves the reliability issues related to real-world applications, which stem from motion artifacts, temporal and postural variability, and the necessity for sensor re-positioning. To extend the practical utility of sEMG-based recognition systems, addressing these challenges is crucial [14]. Variability in sEMG signals presents a challenge that affects the accuracy of hand gesture recognition, proving the importance of going through signal processing and feature engineering. Many sEMG features mentioned in the related research are useful to a certain extent; thus, choosing the most relevant set of features to be extracted is a challenging step itself [15]. The process of selecting an informative and effective set of features is complex by nature, requiring careful consideration and time-consuming feature extraction. The existing literature presents a number of methods for evaluating and selecting features, with the objective of identifying the optimal set of features while retaining the most important information from the sEMG signal. This paper presents two main investigations. The first examines the performance of feature evaluation methods on 13 UCI benchmark datasets in order to test the performance generalization of different feature evaluation methods with variable datasets in terms of dimension and dimensionality. The second study aims to investigate the importance of information derived from the set of selected features, with a focus on sensor placement-dependent feature selection. This is achieved through the analysis of a real-world dataset collected using multichannel sEMG. The objective is to evaluate the extracted features in order to achieve optimal classification performance. This paper is structured as follows. Following the introduction, a section on related research will be presented, followed by a section on methodology, in which the data collection and the six feature evaluation methods used will be explained. The results of the tests with the benchmark dataset and with the real dataset will then be discussed and interpreted in the next section, before the conclusion is reached.

## 2. Related Research

sEMG feature extraction represents a key step in hand gesture classification applications, promoting heightened classification accuracy, efficiency, flexibility, and real-time control, thus increasing the effectiveness of recognizing an extensive array of hand gestures. Different feature evaluation methods are used in the related research. Various approaches such as filter, wrapper, and embedded methods are used for feature selection. Table 1 presents different feature evaluation methods mostly used for sEMG feature extraction in different applications.

The deep learning approach presented in [14] has shown that the variability between subjects, sessions, and arm postures has a significant impact on the system’s accuracy, highlighting the need for the development of strategies to overcome these challenges. A systematic review of sEMG-based classification systems emphasizes the importance of evaluating feature extraction techniques instead of relying on raw sEMG signals to enhance the accuracy of gesture identification [15,27]. The choice of classification algorithm plays a decisive role, as demonstrated by a study achieving an accuracy of 85.7% with pattern recognition techniques [14]. The number of sEMG sensors deployed also emerges as a crucial factor, with a study on forearm amputees revealing its effect on hand posture recognition accuracy [28]. Additionally, the quality of the sEMG signal and the chosen processing method significantly influence classification accuracy, as emphasized by a study employing Convolutional Neural Networks (CNNs) for gesture recognition [29]. Overall, factors affecting the accuracy of sEMG-based hand gesture recognition, even with feature extraction, surround reliability problems, variability, the choice of the classification algorithm, the number of sensors, and signal quality. Addressing these factors is essential for refining the accuracy of sEMG-based hand gesture recognition systems. This feature evaluation process aids in model optimization, as demonstrated by [30]. The model demonstrates how the assessment of extracted features from sEMG signals can optimize the overall classification model. The use of clear, objective features enhances comprehension and facilitates instrumental evaluation. Toledo et al. (2024) extracted a set of 34 time-domain features from sEMG signals collected from four different muscles. The paper’s focus is on selecting the best sEMG feature set for a highly accurate classification application with less computational cost, using the Fisher score method [31]. Our dataset was used to test the Fisher score method, and the results showed that it did not select a set of features that provided good classification performance, despite being a fast feature selection method.

## 3. Methodology

**Figure 1:**
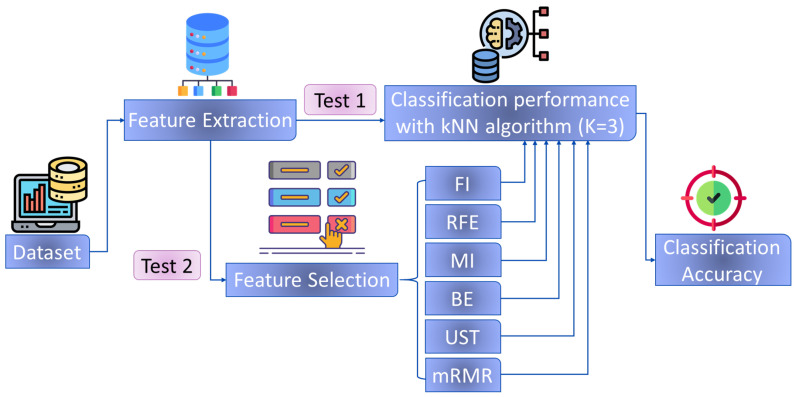
Flowchart of classification performance evaluation with and without feature evaluation methods.

The diagram in the flowchart in Figure 1 presents the methodology employed in both investigations. Two principal tests were conducted for each dataset. Initially, the classification accuracy was evaluated using the original data without feature selection. The performance of the kNN algorithm (K = 3) was noted, and each original feature was subsequently evaluated using the six distinct evaluation methods. Following feature selection, the classification algorithm’s performance was once again tested. Following the evaluation of the selected features, the performance of the kNN algorithm was investigated by examining the identification accuracies of each hand force exercise. This allows for a more comprehensive and detailed analysis of the classification accuracies.

### 3.1. Feature Evaluation Methods

Feature selection is categorized into three main types, which are filter, wrapper, and embedded feature selection. In the context of feature selection, a filter method evaluates the relevance of each feature independently of the others, while a wrapper method uses a specific machine learning algorithm to evaluate the usefulness of a subset of features. In this paper, we are concerned with wrapper and filter feature selection methods, which are as follows:

- **Mutual Information (MI):** The MI method objectively quantifies the dependence between two variables. This measure determines the amount of information that can be gained about one variable from another in feature selection [32]. The objective of MI-based feature selection is to maximize the mutual information between the selected feature subset and the target variable. Mutual Information can be used as both filter and wrapper feature selection methods. In this paper, we implemented MI as a wrapper method from the sklearn Python library based on the entropy estimation metric from k-Nearest Neighbor (kNN) distances.
- **Univariate Statistical Test (UST):** The UST is utilized as a feature selection technique to assess the statistical significance of characteristics. It aims at identifying the most informative and useful set of sEMG features for hand gesture recognition [33]. The UST is a straightforward filter method that evaluates the necessary features to be integrated into the reduced dataset. This method is highly adopted in the fields of machine learning and data mining due to its computational efficiency.
- **Recursive Feature Elimination (RFE):** RFE is a technique for selecting features in which features are chosen sequentially by removing one or a few features at a time in each iteration. The main aim is to choose features recursively by considering them in smaller and smaller subsets. In RFE, an estimator is initially trained using all of the available features, after which the importance of each variable is calculated [34]. RFE is a wrapper-style feature selection algorithm that also uses filter-based feature selection algorithms. In this paper, a wrapper RFE method is implemented based on the Logistic Regression model.
- **Feature Importance (FI):** FI is a technique used to select features that interpret machine learning models constructed from explanatory variables. The main objective of FI is to assess the relative importance of each feature in a dataset when building a predictive model [35]. FI can be implemented as a filter or wrapper method that determines which features should be included in the reduced dataset. This paper uses wrapper-FI based on the Extra Tree Classifier to assess the information value of each element. A score is assigned to show the significance of input constituents toward the algorithm’s decision.
- **Minimum Redundancy Maximum Relevance (mRMR):** mRMR is a method to select a subset of features that are highly relevant to the target variable and have minimal redundancy with each other. The mRMR algorithm ranks features based on their relevance and redundancy scores, where the algorithm selects top-ranked features with the highest relevance and lowest redundancy [36]. The mRMR algorithm is a filter method that evaluates the features to include in the reduced dataset. It has demonstrated effectiveness in selecting the most informative and practical feature set for sEMG-powered hand gesture recognition.
- **Backward Elimination (BE):** BE is a machine learning-based feature evaluation technique that chooses a subset of features from a specified group of features. The process begins by fitting a model with all the independent variables. Next, the variable with the highest p-value is eliminated from the model, and a new model is fitted. This iterative process continues until all model variables exhibit a p-value that falls beneath a predetermined threshold, typically 0.05 [37].

### 3.2. Benchmark Dataset for Feature Evaluation

To generalize the investigation in this paper, we tested each feature evaluation method with thirteen UCI benchmark datasets. We divided the datasets into three groups based on the number of instances, small, medium, and large, as presented in Table 2.

The results of all the tests with the UCI benchmark datasets are presented in Table 3 in the next section.

### 3.3. sEMG Data for Feature Evaluation

**Figure 2:**
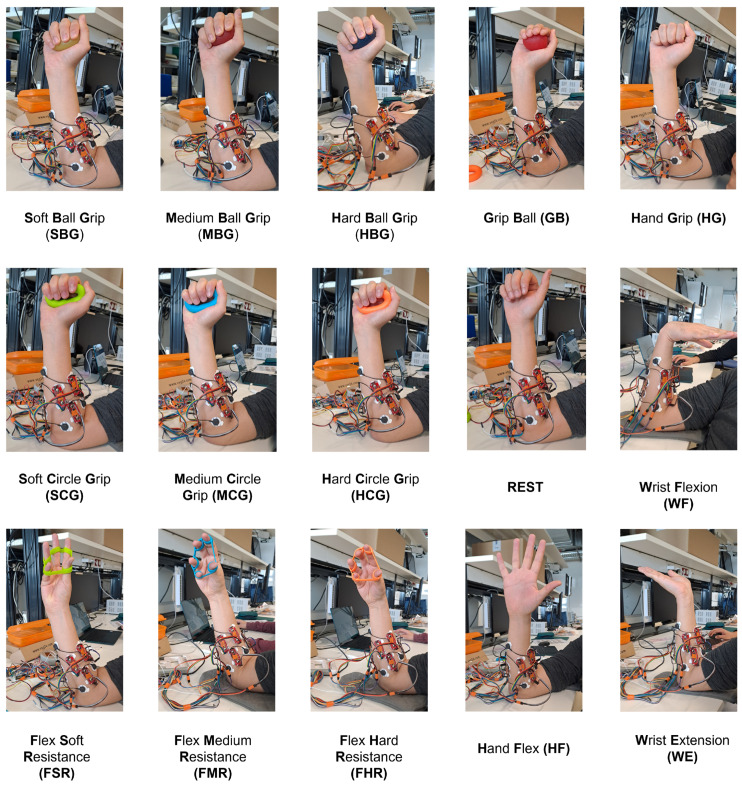
Hand force exercise gestures.

The comprehension of muscle activity during the performance of hand gestures is of significant importance for the diagnosis of muscle during data collection, the facilitation of human–machine interaction, and the development of rehabilitation applications. Hand muscle synergies refer to the coordinated activation patterns of multiple muscles that allow the hand to adopt specific postures or movements. Studies have demonstrated that despite the high number of degrees of freedom in the hand, a reduced set of muscle synergies can account for a significant portion of the variance in hand postures and movements. These synergies represent fundamental building blocks that the central nervous system combines to produce complex hand behaviors. A principal component analysis (PCA) of kinematic data and EMG recordings has revealed that a few principal components or synergies can explain over 80% of the variance in hand postures and muscle activations during grasping tasks [51,52,53]. This suggests the existence of low-dimensional control strategies that simplify the coordination of the many muscles and joints involved in hand function. The identified synergies frequently correspond to common grip types such as power grips or precision grips, as well as finer adjustments for object manipulation [51]. By flexibly combining these synergies, the neuromuscular system can generate a wide repertoire of hand postures and movements required for dexterous object interactions [52,54]. The choice of the set of gestures presented in this paper is based on a variety of hand exercises, wherein the participant is required to apply grasping, flexion, and extension force in order to maintain these gestures, which present varying degrees of difficulty. Consequently, the acquired sEMG signals during the performance of these gestures will provide a clear understanding of the underlying muscle activity, enabling the determination of muscle force and the identification of any fatigue in the muscles. In this direction, a set of 15 hand gestures, inspired by hand force exercises, is defined as shown in Figure 2. The list of gestures contains seven grasping exercises, four hand flexion exercises, wrist extension, and wrist flexion positions. A rest position and a grip ball are the only two non-force gestures in the list. The two positions were selected as reference points for the collection of sEMG signals. The first was the rest position, which was defined as the hand being completely relaxed. The second was the grip ball (GB) gesture, which was defined as the hand holding the ball without exerting any force.

**Figure 3:**
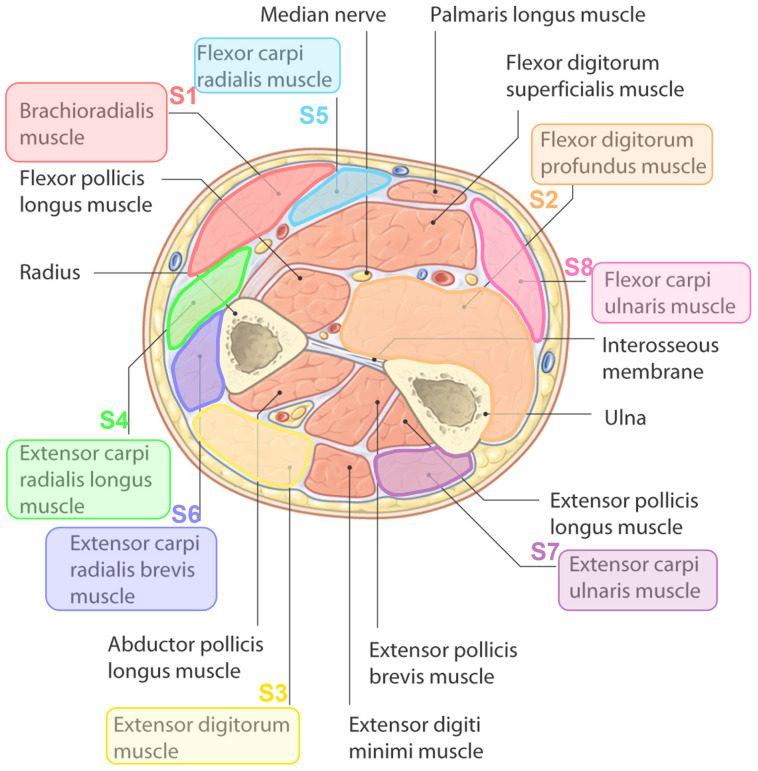
sEMG sensor positions in the forearm muscles.

In order to define the position of the sEMG sensors, a forearm anatomy study was conducted to identify the superficial muscles responsible for maintaining the flexion, extension, and grasping exercises presented in the list. The output of the forearm anatomy study defined a list of eight muscles that participate in maintaining each of the force exercises. These muscles are superficial and can be reached with the sEMG sensors. Consequently, eight MyoWare commercial sEMG channels are positioned on distinct hand muscles, including the brachioradialis, flexor digitorum profundus, extensor digitorum, extensor carpi radialis longus, flexor carpi radialis, extensor carpi radialis brevis, extensor carpi ulnaris, and flexor carpi ulnaris muscles. This is illustrated in Figure 3. The aforementioned hand muscles have been identified as those most essential for each of the aforementioned hand exercises.

This set of hand force exercises was collected from 14 healthy participants (6 males and 8 females) with an average age and Body Mass Index (BMI) of 25.14 ± 2.8 years old and 24.81 ± 3.6 kg/$m^{2}$, respectively. In order to record the sEMG signal under more realistic conditions, data collection was performed while all the participants were fasting from food and coffee [55]. To ensure robust data for the machine learning-based gesture classification, each participant performed every gesture 20 times. The trials were structured with participants instructed to perform the target gesture for a duration of 4 s, allowing for the capture of 2000 data points from each surface electromyography (sEMG) channel sampled at 500 Hz. After each four-second gesture execution, the participants were given a five-second rest interval to minimize muscle fatigue and ensure clear delineation between gesture repetitions. By adhering to this structured data collection protocol, a comprehensive dataset was compiled, consisting of 20 repetitions for each gesture across all participants. The 4 s window and 500 Hz sampling rate provided a high-resolution temporal representation of the muscle activation patterns, enabling the machine learning models to effectively extract features and learn and classify the gestures based on the sEMG data.

**Figure 4:**
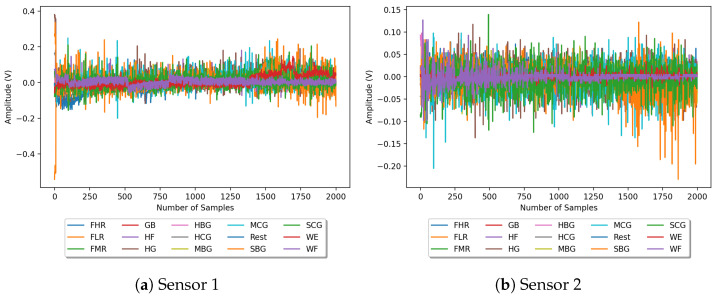

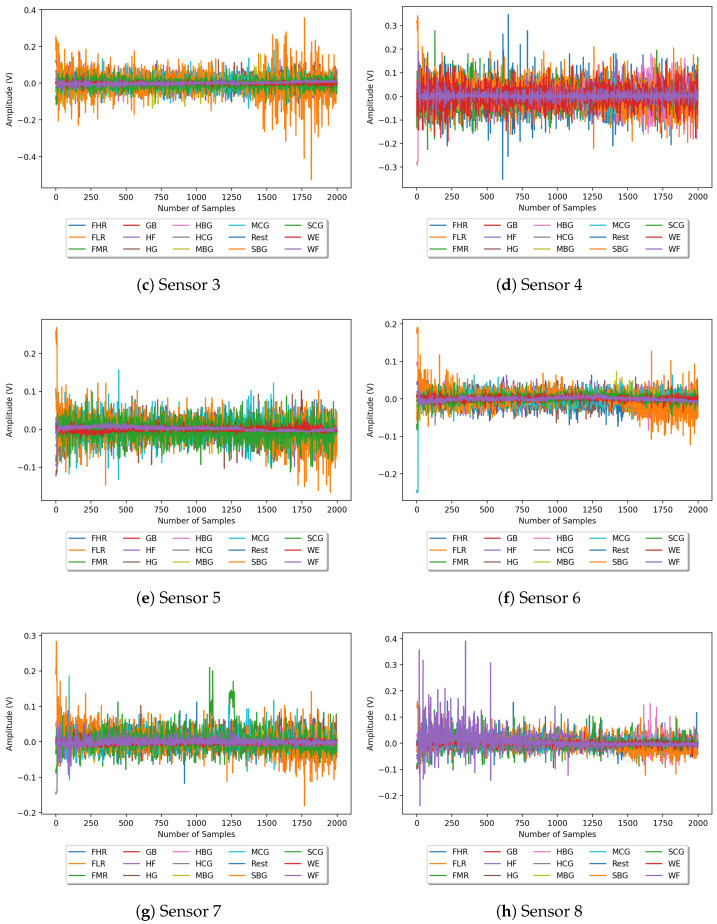
Raw signals recorded using sEMG sensors during hand force exercises for 4 s measurement.

Figure 4 shows the eight raw sEMG signals recorded from one of the participants while performing the fifteen hand force gestures. The acquired signals were filtered from the commercial sEMG sensors, which employ a differential amplifier and an analog bandpass filter (200–500 Hz) to amplify and filter the raw sEMG signal. Moreover, for the purpose of extracting features, it is preferable to utilize the raw shape of the signals rather than the rectified version. A total of 37 time- and frequency-domain features are extracted from each sEMG channel, as defined in Table 4. These features were collected from various research papers working with sEMG signals. Certain features, such as the MAV, WL, RMS, SSC, ZC, VAR, SSI, MMAV1, and MMAV2, are more frequently used in the literature. Moreover, sEMG signal features such as the MaxAV, MinAV, RSSQ, and P2P are not frequently utilized in the related research. The majority of the extracted features in the list are time-domain features, which have the advantage of being more straightforward to compute and requiring less computational resources. Consequently, they can be employed in real-time applications. Furthermore, they have been demonstrated to achieve high gesture recognition accuracy (exceeding 90%) when combined with machine learning classifiers [56,57]. Nevertheless, time-frequency-domain features, such as Short-Time Fourier Transform (STFT) coefficients, Spectral Moment (SM), and Stockwell Transform coefficients, have the potential to capture non-stationarities in sEMG signals. However, studies have not conclusively demonstrated superior performance over time-domain features, which would justify the added computational complexity for many applications [56,58,59]. After extracting 37 features from each of the eight sEMG channels, we tested the performance of the kNN classification algorithm using the database containing 296 features (37 × 8 = 296). The kNN algorithm achieved a validation accuracy of up to 95.83% without feature selection. In the subsequent session, we evaluated the performance of the kNN after undergoing the feature evaluation step.

## 4. Results and Discussion of Feature Evaluation Methods

The analysis of the feature evaluation conducted across the 13 UCI benchmark datasets, as detailed in Table 3, shows the effectiveness of various feature selection methods in enhancing classification accuracy in comparison with the original classification accuracy. Recursive Feature Elimination (RFE), Feature Importance (FI), the Univariate Selection Test (UST), and Mutual Information (MI) are shown to consistently improve accuracy across diverse datasets. Conversely, the mRMR method does not significantly enhance classification accuracy in most cases and may even decrease it.

On the other hand, this observation is particularly compelling for certain datasets, such as Iris. The datasets demonstrate remarkable resilience in maintaining high classification accuracy, even when only two out of the four features are selected, regardless of the feature evaluation method used. This finding suggests that certain datasets, such as the well-studied Iris dataset in the UCI benchmark, inherently possess an optimal set of features that robustly contribute to accurate classification. The reliable performance of Iris across various feature selection methods suggests that there may be some redundancy or interdependence among the features, highlighting the distinctive characteristics of the dataset that make it ideal for classification tasks. This understanding of dataset-specific behaviors provides valuable insights into the broader field of feature selection and classification. It offers guidance on optimal feature exploitation for improved model performance across different datasets.

**Figure 5:**
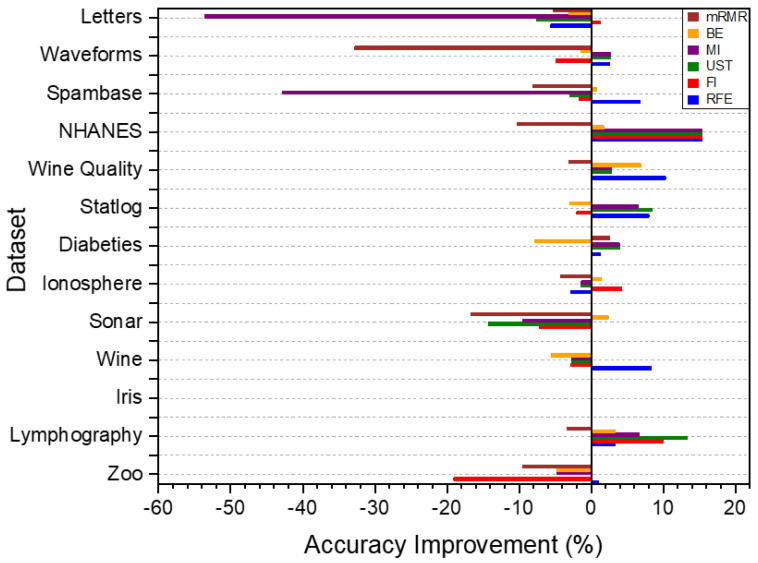
Classification accuracy improvement for the benchmark dataset after feature selection.

Figure 5 presents the accuracy improvements for all the feature evaluation methods with each benchmark dataset. The accuracy improvement is calculated by subtracting the original accuracy value from the new accuracy value, obtained from the classification test with the new data subset after feature selection.

The Recursive Feature Elimination (RFE) method and the Mutual Information (MI) method both show positive accuracy improvement values in almost all the dataset cases. However, the MI method has a limitation of decreasing classification accuracy values with a high negative accuracy improvement in some datasets, such as the Letters and Spambase datasets, with a degradation equal to −53.65% and −42.80%, respectively.

**Figure 6:**
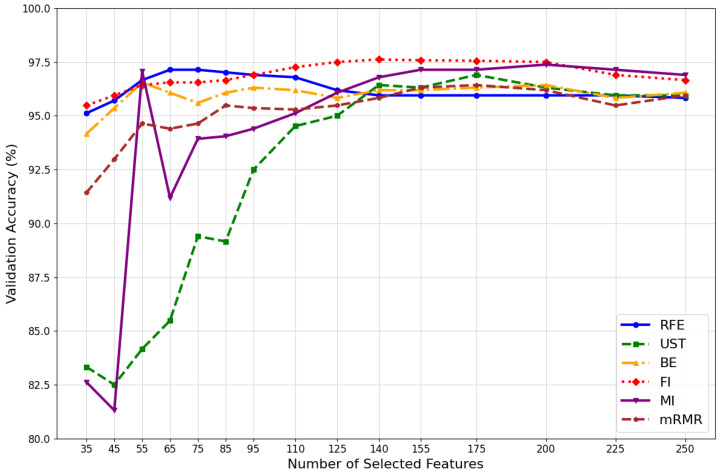
Comparison of validation accuracy of feature selection methods on sEMG dataset.

To evaluate the reliability of each feature selection method, we tested the classification algorithm’s performance to determine the set of features that resulted in the highest validation accuracy when classifying the 15 hand rehabilitation exercises. We conducted 15 tests for each of the six feature evaluation methods, as shown in Figure 6. For each test, we selected the ‘n’ best features from the output of each method and evaluated the classification performance. The value of ‘n’ ranged from 35 to 250. Notably, the accuracy values were sub-optimal when the size of the feature set was very small, such as for a set of 35 or 45 features, or when the size was larger than 200 features, as in the case of the UST, FI, mRMR, and MI methods.

Figure 6 shows that the top four feature valuation methods are FI, MI, RFE, and the UST. The highest classification accuracies, up to 97.62%, 97.38%, 97.14%, and 96.90%, respectively, were achieved with sets of 140, 200, 65, and 175 selected features. It can be concluded that the RFE method provides better classification accuracy than the original method, reducing the original feature size from 296 to only 75 features. The difference between these feature evaluation methods lies in their approach to identifying and eliminating useful and irrelevant features. The MI and FI methods require a set of 200 and 140 features, respectively, to achieve optimized classification accuracy. This highlights the weakness of these methods in distinguishing between necessary and redundant features.

Despite the higher computational costs of wrapper feature selection methods compared to standard ones, they have been demonstrated to be a superior approach. The iterative aspect of wrapper methods allows them to explore feature subsets in a systematic way, adding or removing features based on their impact on model performance. This approach has been demonstrated to outperform standard methods such as information gain and Chi-squared tests across a variety of datasets and learning algorithms, as well as the Forward Feature Selection (FFS) [72] and the Sequential Floating Feature Selection (SFFS) [73]. Moreover, wrappers are capable of capturing feature dependencies and interactions that filters are unable to identify, resulting in enhanced predictive performance [74]. The iterative wrapper approach is also more computationally efficient than evaluating all possible subsets, which is infeasible for even moderate numbers of features [75].

It can be assumed that the addition of further features to the input dataset provided to the machine learning model would result in an improvement in classification accuracy. However, this is not always the case, as evidenced by the results of this test. In fact, there are instances where the addition of further data or features can actually increase the ambiguity between the classes, thereby decreasing the classification accuracy. This phenomenon was observed in all feature evaluation cases while increasing the size of the set of selected features. One key reason for this is the concept of the curse of dimensionality. As the number of features in a dataset increases, the model must learn patterns in a much higher-dimensional space. This can make it more difficult for the model to generalize effectively, as it becomes harder to find the most relevant signals amidst the noise. Furthermore, the risk of overfitting also increases with the addition of more features. This occurs when the model memorizes the training data too closely and fails to generalize well to new, unseen examples. Another factor is the quality and relevance of the additional information being added. If the new features are not truly informative or are redundant with existing features, they may not provide any meaningful signal information to the model. In some cases, irrelevant or noisy features can even confuse the model and degrade its performance. To provide useful information from each of the eight signals, it is important to evaluate the significance of each feature and its role. Therefore, we investigated the set of features that yielded the highest classification accuracies for the RFE, FI, and MI methods. Table 5 presents the channels from which each feature is extracted and selected after evaluation with the RFE, MI, and FI methods with the set of features giving the highest classification accuracies.

Among the various features extracted from the surface electromyography (sEMG) signals, a subset of features, including the skewness, mean frequency (MNF), mean median frequency (MDF), and kurtosis, demonstrate limited utility as they either remain unselected by any channel or are chosen by only a single channel. This lack of consistent selection across channels highlights their limited ability to extract significant information from the signals, emphasizing their potential exclusion from the feature set. These features were consistently selected across different channels in all three feature evaluation cases. A subset of features, including the Simple Square Integral (SSI), Slope Sign Change (SSC), Willison Amplitude (WAMP), Waveform Length (WL), Average Signal Energy Ratio (AVSER), and Average Squared Signal Energy Ratio (AVSSR), among others, exhibit noteworthy significance. The inclusion of these features across multiple channels emphasizes their relevance and effectiveness, positioning them as high-priority options for feature extraction in the context of signal processing and classification tasks. Their consistent selection across diverse channels highlights their robustness and potential to capture essential information encoded in sEMG signals, making them valuable contributors to accurate and comprehensive gesture classification systems.

The validation accuracy test with the selected features using the RFE method revealed that the kNN algorithm achieved the highest classification accuracy, equal to 97.14%, in two cases. The first case involved the set of 65 features, and the second case involved the set of 75 features. Table 6 presents a comparison of the selected features in the two cases, with the RFE method. The 10 additional features included in the list of 75 features, in addition to the list of 65 features, comprise 3 features for sensor 4 (MAD0, AVSER, and LD), 2 features for sensor 5 (MAD1 and AVSSR), and only 1 feature for the other sensors, with the exception of sensor 3.

**Figure 7:**
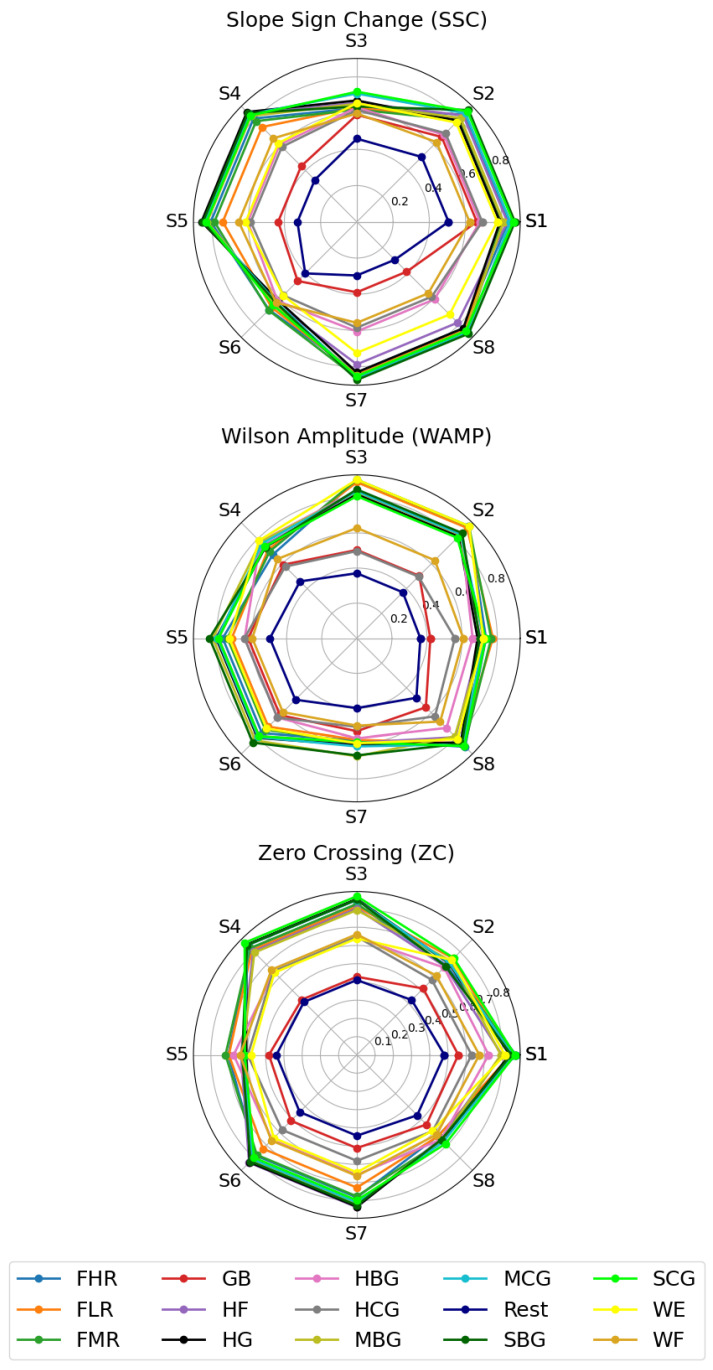
Radar plot of the top selected features with RFE method.

The findings of this study indicate that the features Slope Sign Change (SSC), Wilson Amplitude (WAMP), and Zero Crossing (ZC) were identified as the top three features selected from all eight sensors in both cases. To gain a deeper understanding of the rationale behind the significance of these three features, Figure 7 presents a radar plot of each feature across the eight sensors and 15 hand force exercises. The radar plots provide valuable insights into the discriminative power of these features. The SSC feature exhibits clear differences in its patterns across the 15 classes, indicating its ability to differentiate between the hand force exercises. Similarly, the WAMP feature exhibits distinct profiles for the various classes, suggesting that it captures important information related to the hand force activities. In other research, the WAMP was selected in the optimal feature subsets across different subjects, suggesting its suitability for representing forearm sEMG signals and recognizing gestures [57,76]. The ZC feature also shows unique signatures for each of the 15 classes, highlighting its relevance in extracting meaningful data from the sensor channels. The visualization of the features in a multi-dimensional radar format allows for a more comprehensive understanding of the reasons behind the Recursive Feature Elimination (RFE) method’s selection of these three characteristics as the most informative for the classification task. The distinct shapes and positions of the data points on the radar plots demonstrate the rich, discriminative information contained within the SSC, WAMP, and ZC features across the eight sensor channels. This visual analysis serves to corroborate the importance of these features in achieving high classification performance for the 15 hand force exercises. Their importance is related to their complementarity in providing precise information about the classes. As an example, in the radar plot of the ZC feature, we can see the differentiation of the values from one class to another, particularly with sensors 1, 6, and 7, which is not the case with sensors 3 and 4. The role of the other features, such as the SSC with sensor 4 and the WAMP with sensor 3, is also evident. These features demonstrate higher differentiation between the classes, which corroborates the complementarity between these features.

**Figure 8:**
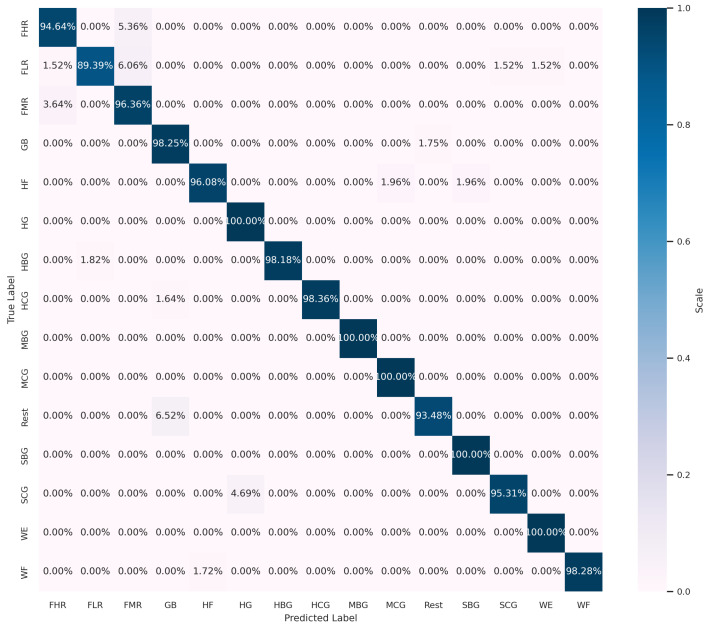
Confusion matrix with 65 selected features by RFE.

**Figure 9:**
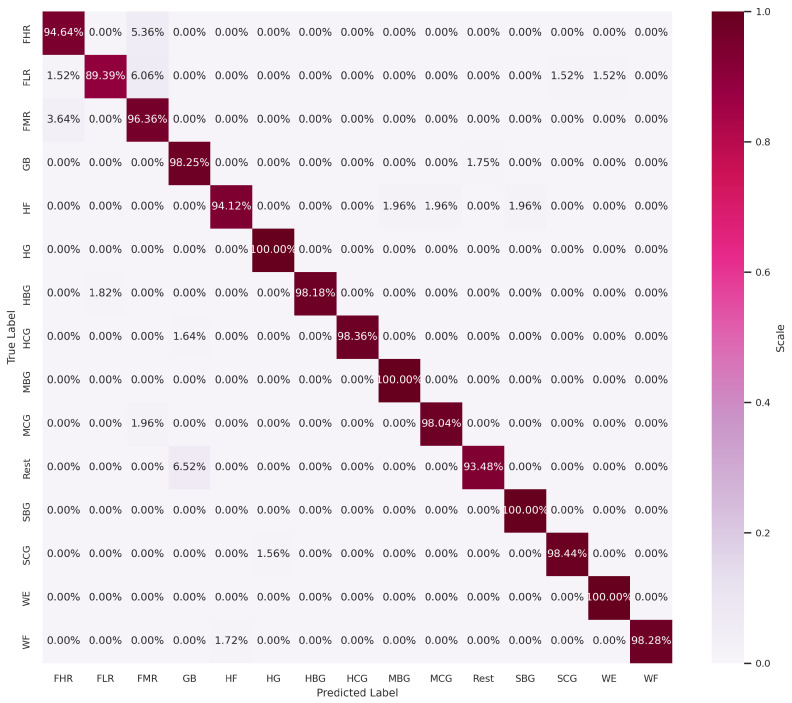
Confusion matrix with 75 selected features by RFE.

**Figure 10:**
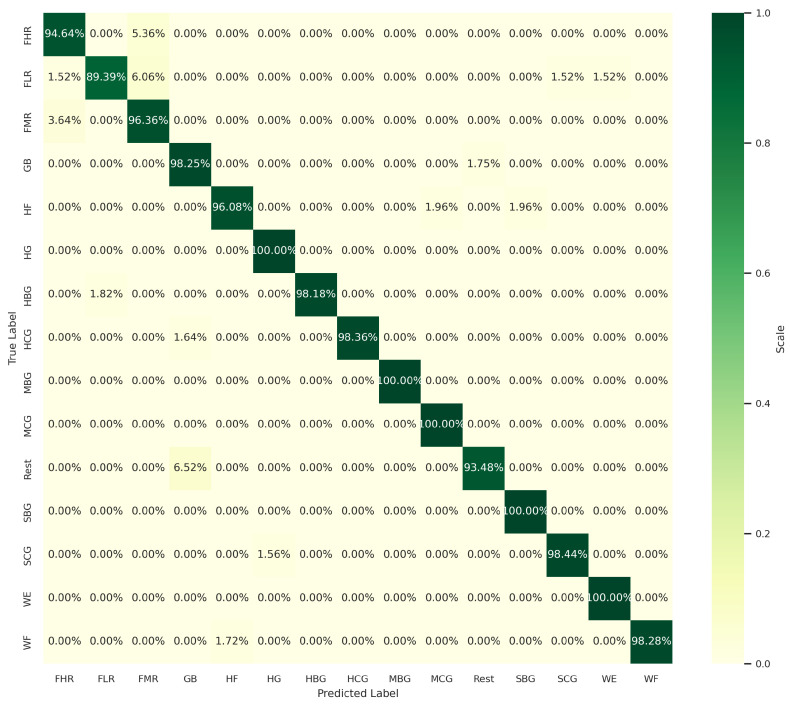
Confusion matrix with 68 selected features by RFE.

To gain further insight into the impact of these ten additional features on classification accuracy, we compare the confusion matrix outputs in both cases. As shown in Figure 8 and Figure 9, the identification accuracy of all the classes remains the same in both cases, with the exception of three classes. The classification accuracies of the exercises HF and MCG exhibited a decline from 96.08% to 94.12% and from 100% to 98.04%, respectively. In contrast, the accuracy of the SCG exercise demonstrated an increase from 95.31% to 98.44%. The stability of the classification accuracy in both cases does not imply that all the classes are identified with equal precision. In this case, the stability of the accuracy is due to an increase in the identification of one class and a decrease in the identification of two other classes. In order to investigate the role of the features that have a role in increasing the identification accuracy of the SCG exercise, an experiment was conducted to test the impact of certain features, added from the ten extra features of the list of 75, on the classification performance. Based on a strategic feature selection from the set of ten extra features, a different set of features was selected for each test. The strategy is based on prioritizing the addition of features extracted from sensors placed on the group of muscles responsible for hand grasping exercises, including sensors 1, 2, 3, 4, 5, and 7. We first excluded the TM(5) and MVSR features extracted from sensors 6 and 8, respectively. This exclusion had no effect on the classification accuracy. This is a logical conclusion, as the two sensors in question are not included in the list of sensors positioned to monitor grasping. We then began to test the impact of each of the remaining eight features in the list on the classification performance. The results of these tests show that the features AVSER, AVSSR, and MVSR, extracted from sensors 4, 5, and 7, respectively, have a high impact on increasing the validation accuracy to 97.38%. As seen in Figure 10, the identification accuracies of the HF and MCG classes remain the same at 96.08% and 100%, respectively, as with the set of 65 features. And for the SCG exercise, the identification accuracy is equal to 98.44%, as in the case of using the set of 75 features.

## 5. Conclusions

In conclusion, this study emphasizes the importance of effective feature extraction and selection for the success of human–machine interaction applications, especially in accurately classifying and predicting hand gestures. This research systematically compared six filter and wrapper feature evaluation methods, providing valuable insights into their respective impacts and paving the way for improved gesture recognition systems. This study presents experimental results based on a diverse benchmark dataset and a real hand gesture dataset. It offers a comprehensive understanding of the strengths and limitations of the evaluated techniques. This study involved 14 healthy participants who performed 15 hand force exercises using eight MyoWare sEMG sensors placed on various muscles in the forearm. From each sEMG channel, 37 time- and frequency-domain features were extracted. This study shows that the Recursive Feature Elimination (RFE), Mutual Information (MI), and Feature Importance (FI) feature evaluation methods perform better, resulting in feature subsets with higher classification accuracies of up to 97.38%, 97.38%, and 97.62%, respectively, with different sets of features. A detailed examination of the selected features is essential to gain a comprehensive understanding of their impact on the classification accuracy. In the case of RFE, 65 and 75 features yielded an identical classification accuracy of 97.14%, while the addition of only 3 features to the 65-feature set resulted in a 97.38% classification accuracy. These findings highlight the potential of specific feature evaluation methods to enhance the discriminative power of selected features, contributing to the development of more robust and accurate hand gesture recognition systems. This study employed diverse datasets, including both benchmark and real-world scenarios, which adds credibility to the generalizability of the results. Overall, this research advances our understanding of feature selection methodologies in the context of sEMG-based gesture recognition, providing practical insights for the design and optimization of human–machine interaction systems.

## Figures and Tables

**Table 1:** Feature evaluation methods in the related research.

Type	Feature Selection Method	Used Dataset	Application
**Filter**	Pearson Correlation		
	Chi-squared Test	2-channel sEMG	9 hand gestures recognition [16]
	Relief Feature Selection (Relief-F)		
	Univariate Statistical Test (UST)	Multichannel ECG	Stress detection during job interview [17]
	Maximum Relevance Minimum Redundancy (MRMR)	8-channel sEMG	7 hand gestures classification [18]
**Wrapper**	Recursive Feature Elimination (RFE)	3-axis accelerometer and 9-channel sEMG	Gender recognition in normal walking [19]
	Backward Elimination and Forward Selection	Cleveland Heart Disease dataset	Heart disease prediction [20]
	Feature Importance (FI) based on Extra Tree Classifier	Stress detection prediction Kaggle dataset	Stress prediction [21]
	Mutual Information (MI)	Multichannel sEMG	Hand movement recognition [22]
	Swarm Intelligence Algorithms	2-channel sEMG	American signs classification [23,24]
**Embedded**	Least Absolute Shrinkage and Selection Operato (LASSO)	EEG and EMG channels	Healthcare monitoring system [25]
	Regularized Regression Models	24 classification datasets	Classification [26]

**Table 2:** UCI benchmark datasets.

Size	Dataset Name	#Features	#Instances	#Classes
	Zoo [38]	16	101	7
	Lymphography [39]	18	148	4
Small	Iris [40]	4	150	3
	Wine [41]	13	178	3
	Sonar [42]	60	208	2
Medium	Ionosphere [43]	34	351	2
	Diabetes [44]	8	768	2
	Statlog [45]	20	1000	2
	Wine Quality [46]	11	1599	11
Large	NHANES [47]	9	2278	2
	Spambase [48]	57	4601	2
	Waveform [49]	40	4962	3
	Letter [50]	16	20,000	26

**Table 3:** Feature evaluation results with UCI benchmark datasets.

Size	Dataset	Original Accuracy (%)	Original Number of Features	RFE		FI		UST		MI		BE		mRMR	
				Accuracy (%)	NB of Feat	Accuracy (%)	NB of Feat	Accuracy (%)	NB of Feat	Accuracy (%)	NB of Feat	Accuracy (%)	NB of Feat	Accuracy (%)	NB of Feat
Small	Zoo	95.24	16	96.24	08	76.19	03	95.24	09	90.48	09	90.48	08	85.71	02
	Lymphography	73.33	18	76.67	09	83.33	04	86.67	06	80.00	06	76.67	10	70.00	02
	Iris	93.33	04	93.33	02	93.33	02	93.33	02	93.33	02	93.33	02	93.33	02
	Wine	83.33	13	91.66	06	80.55	08	80.56	10	80.56	10	77.78	09	83.33	10
	Sonar	78.57	60	78.57	30	71.43	04	64.29	05	69.05	05	80.95	47	61.90	05
Medium	Ionosphere	84.51	34	81.69	17	88.73	04	83.10	08	83.10	09	85.92	22	80.28	08
	Diabetes	67.53	08	68.83	04	67.53	01	71.43	04	71.43	04	59.74	04	70.13	04
	Statlog	62.00	20	70.00	10	60.00	09	70.50	02	68.50	02	59.00	14	62.00	02
	Wine Quality	49.38	11	59.69	05	49.38	03	52.19	02	52.19	02	56.25	07	46.25	02
Large	NHANES	84.65	09	100	04	100	01	100	01	100	01	86.40	06	74.34	01
	Spambase	84.15	57	90.88	29	82.41	13	81.22	12	41.35	13	84.91	42	76.11	12
	Waveforms	80.20	47	82.70	20	75.30	05	82.90	11	82.90	13	78.80	23	47.4	11
	Letters	95.00	16	89.35	08	96.28	11	87.45	07	41.35	07	91.95	12	89.75	07

**Note:** The cells highlighted in green represent the highest accuracy achieved for each dataset among the different feature selection methods.

**Table 4:** Definition of the time-domain extracted features.

Features	Definition
Kurtosis (Kurt) [60]	$Kurt=[\frac{\frac{1}{N}\sum_{i=1}^{N}y_{i}^{4}}{{\left(\frac{1}{N}\sum_{i=1}^{N}y_{i}^{2}\right)}^{2}}]-n$
Skewness (Skew) [60]	$Skew=\frac{\frac{1}{N}\sum_{i=1}^{N}{\left(y_{i}-\bar{y}\right)}^{3}}{{\left(\sqrt{\frac{1}{N}\sum_{i=1}^{N}{\left(y_{i}-\bar{y}\right)}^{2}}\right)}^{3}}$
Simple Square Integral (SSI) [61]	$SSI=\sum_{i=1}^{N}x_{i}^{2}$
Root Mean Square (RMS) [61]	$RMS=\sqrt{\frac{1}{N}\sum_{i=1}^{N}x_{i}^{2}}$
Shannon Entropy [62]	$En\left(x\right)=-\sum_{i=1}^{N}P_{i}\log\left(P_{i}\right)$, where *x*: discrete random variable, $x_{i}\in \left\{x_{1},\ldots ,x_{N}\right\}$, probabilities $P_{i}\in \left\{P_{1},\ldots ,P_{N}\right\}$
Mean Absolute Value (MAV) [61]	$MAV=\frac{1}{N}\sum_{i=1}^{N}\left|x_{i}\right|$
Average Amplitude Change (AAC) [63]	$AAC=\frac{1}{N}\sum_{i=1}^{N-1}\left|x_{i+1}-x_{i}\right|$
Difference Absolute Standard Deviation Value (DASDV) [63]	$DASDV=\sqrt{\frac{\sum_{i=1}^{N-1}{\left(x_{i+1}-x_{i}\right)}^{2}}{N-1}}$
Log Detector (LD) [63]	$exp(\frac{1}{N}\sum_{i=1}^{N}\log\;\left|x_{i}\right|)$
Modified Mean Absolute Value 1 (MMAV1) [63]	$MMAV1=\frac{1}{N}\sum_{i=1}^{N}\omega_{i}\left|x_{i}\right|$, with $w_{i}=\left\{\begin{array}{cc} 1, & 0.25L\leq i\leq 0.75\\ 0.5 & \mathrm{otherwise} \end{array}\right.$
Modified Mean Absolute Value 2 (MMAV2) [63]	$MMAV2=\frac{1}{N}\sum_{i=1}^{N}\omega_{i}\left|x_{i}\right|$, with $w_{i}=\left\{\begin{array}{cc} 1, & 0.25L\leq i\leq 0.75\\ \frac{4i}{L} & \mathrm{if}\;i<0.25L\\ \frac{4/i_{L}}{L} & \mathrm{otherwise} \end{array}\right.$
Slope Sign Change (SSC) [64]	$SSC=\sum_{i=2}^{L-1}\left[f\left[\left(x_{i}-x_{i1}\right)\times \left(x_{i}-x_{i+1}\right)\right]\right]$
	with $f\left(x\right)=\left\{\begin{array}{cc} 1, & \mathrm{if}\;x\geq \mathrm{threshold}\\ 0, & \mathrm{otherwise} \end{array}\right.$
Wilson Amplitude (WAMP) [65]	$WAMP=\sum_{i=1}^{N-1}u(|x_{i+1}-x_{i}\left|-T\right)$
Waveform Length or Wavelength (WL) [63]	$WL=\sum_{i=2}^{N}\left|x_{i}-x_{i-1}\right|$
Variance (VAR) [66]	$VAR=\frac{1}{N+1}\sum_{i=1}^{N}x_{i}^{2}$
Root Sum of Square Level (RSSQ) [67]	$RSSQL=\sqrt{\sum_{i=1}^{L}{\left|x_{i}\right|}^{2}}$
Mean Frequency (MNF) [68]	$MNF=\frac{\sum_{i=1}^{N}f_{i}p_{i}}{\sum_{i=1}^{N}p_{i}}$ where $f_{i}$ is the frequency variable, and $p_{i}$ is the power spectrum
Median Frequency (MDF) [68]	$MDF=\frac{1}{2}\sum_{i=1}^{N}p_{i}$
Peak Frequency (PKF) [68]	PKF = max($p_{i}$), i = 1, …, N
Peak2peak (P2P) [68]	P2P = max(x) − min(x)
Band Power (BP) [68]	Returns the average power in the input signal x, and if x is a matrix, then band power computes
	the average power in each column independently
Temporal Moment (TM) [61]	$TM=|\frac{1}{N}\sum_{i=1}^{N}x_{i}^{order}|$
V-Order (V0) [61]	$VO=(\frac{1}{N}\sum_{i=1}^{N}x_{i}^{\frac{1}{\mathrm{order}}})$
Mean Absolute Deviation (MAD0) [68]	$MAD0=\frac{1}{N}\sum_{i=1}^{N}\left|x_{i}-\mathrm{mean}\left(x\right)\right|$
Median Absolute Deviation (MAD1) [68]	$MAD1=\frac{1}{N}\sum_{i=1}^{N}\left|x_{i}-\mathrm{median}\left(x\right)\right|$
Integrated EMG (IEMG) [68]	$IEMG=\sum_{i=1}^{N}\left|x_{i}\right|$
Maximum of Absolute Value (MaxAV)	$MaxAV=\max(|x_{i}|)$
Zero Crossing (ZC) [69]	$sgn\left(x\right)=\left\{\begin{array}{cc} 1, & \mathrm{if}\;x\geq \mathrm{threshold}\\ 0, & \mathrm{otherwise} \end{array}\right.$,
	$ZC=\sum_{i=1}^{L-1}|x_{i}\times x_{i+1}\left|\cap \right|x_{i}-x_{i+1}|\;\geq \mathrm{threshold}$
Absolute Value of the Summation of Exp Root (AVSER)	$AVSER=\sum_{i=1}^{L-1}\left|{\left(x_{i}\right)}^{\frac{1}{e}}\right|$
Absolute Value of the Summation of Square Root (AVSSR) [70]	$AVSSR=\sum_{i=1}^{L-1}\left|{\left(x_{i}\right)}^{\frac{1}{2}}\right|$
Coefficient of Variance (COV) [71]	The ratio of the standard deviation to the mean (%)
Difference Absolute Mean Value (DAMV) [63]	$DAMV=\frac{1}{N-1}\sum_{i=1}^{N}\left|x_{i+1}-x_{i}\right|$
Interquartile Range (IQR) [68]	$IQR=\mathrm{Median}\left(upper\left(x_{i}\right)\right)-\mathrm{Median}\left(lower\left(x_{i}\right)\right)$
Mean Value of the Square Root (MVSR) [70]	$MVSR=\frac{1}{L}\sum_{i=1}^{L-1}{\left(x_{i}\right)}^{\frac{1}{2}}$
Minimum of Absolute Value (MinAV)	$MinAV=\min(|x_{i}|)$

**Table 5:** Selected features by channels.

Features	Channels by RFE	Channels by FI	Channels by MI
Kurtosis	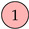	∅	∅
Skewness	∅	∅	∅
SSI	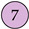	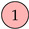 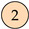 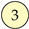 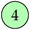 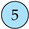 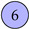 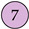 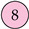	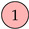 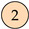 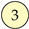 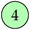 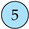 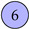 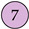 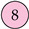
SD	∅	∅	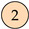 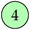 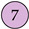
RMS	∅	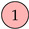 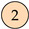 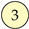 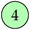 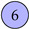 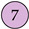 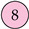	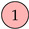 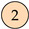 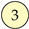 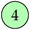 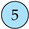 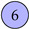 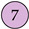 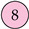
Entropy	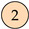 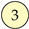 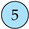 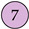	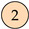 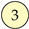 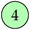 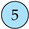 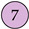 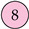	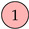 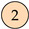 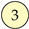 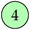 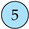 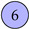 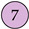 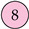
MAV	∅	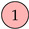 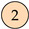 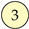 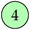 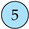 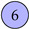 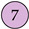 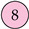	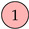 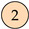 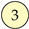 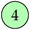 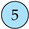 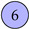 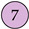 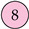
AAC	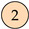 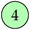 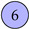 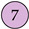	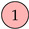 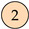 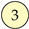 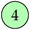 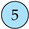 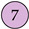	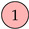 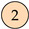 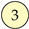 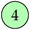 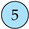 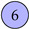 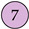 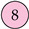
DASDV	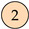	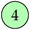	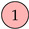 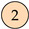 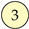 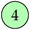 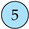 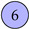 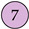 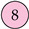
LD	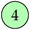 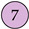	∅	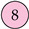
MMAV1	∅	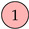 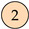 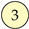 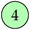 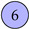 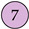 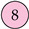	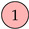 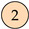 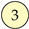 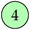 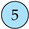 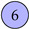 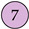 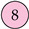
MMAV2	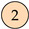 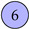	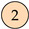 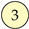 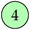 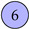 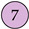 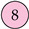	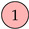 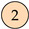 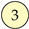 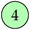 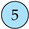 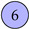 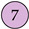 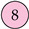
SSC	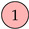 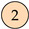 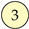 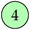 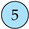 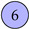 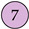 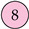	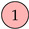 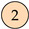 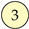 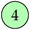 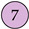 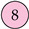	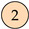 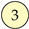 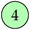 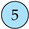 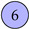 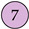 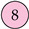
WAMP	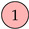 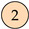 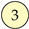 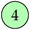 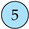 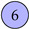 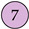 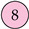	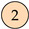 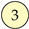 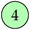 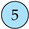 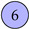 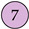 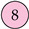	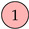 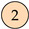 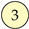 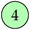 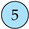 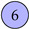 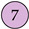 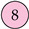
WL	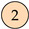 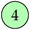 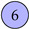 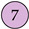	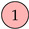 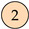 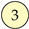 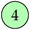 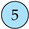 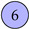	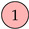 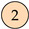 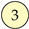 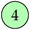 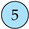 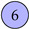 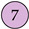 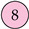
VAR	∅	∅	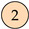 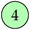 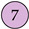
RSSQ	∅	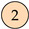 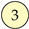 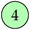 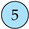 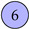 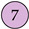 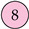	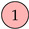 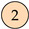 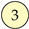 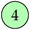 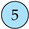 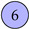 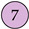 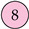
MNF	∅	∅	∅
MDF	∅	∅	∅
PKF	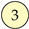 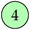	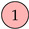 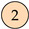 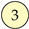 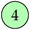 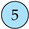 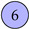 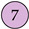 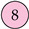	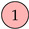 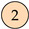 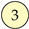 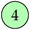 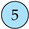 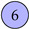 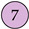 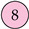
P2P	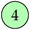	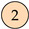 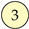 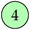 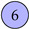 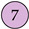 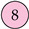	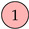 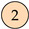 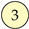 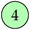 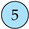 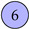 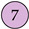 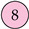
BP	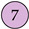	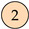 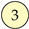 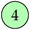 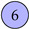 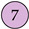 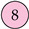	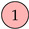 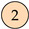 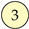 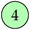 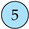 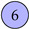 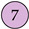 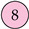
TM(3)	∅	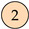 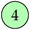 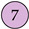 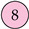	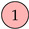 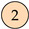 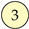 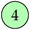 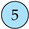 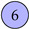 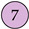 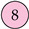
TM(5)	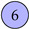	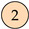 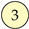 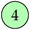 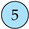 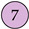	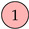 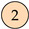 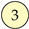 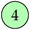 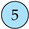 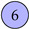 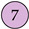 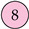
V0	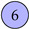	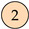 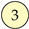 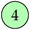 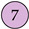 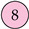	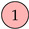 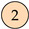 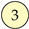 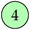 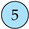 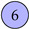 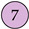 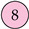
MAD0	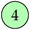	∅	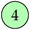
MAD1	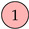 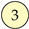 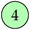 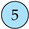	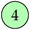	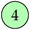
IEMG	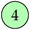	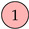 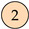 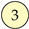 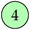 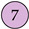 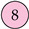	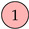 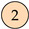 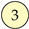 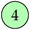 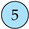 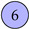 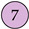 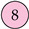
MaxAV	∅	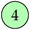	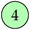 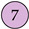
ZC	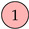 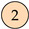 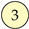 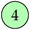 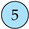 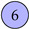 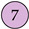 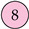	∅	∅
AVSER	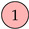 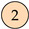 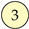 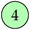 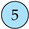 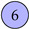 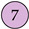	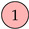 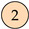 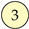 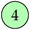 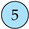 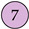 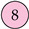	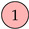 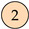 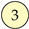 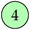 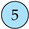 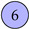 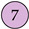 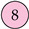
AVSSR	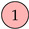 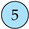 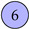 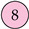	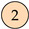 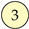 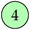 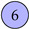 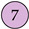 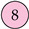	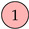 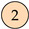 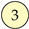 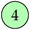 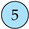 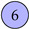 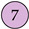 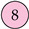
COV	∅	∅	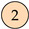 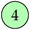 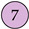
DAMV	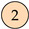 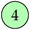 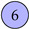 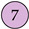	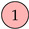 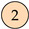 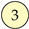 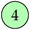 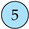 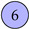 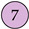 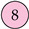	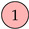 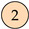 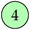 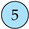 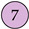
IQR	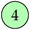	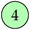	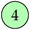
MVSR	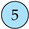 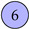 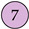 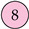	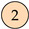 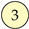 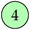 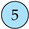 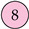	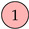 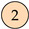 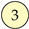 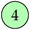 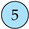 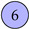 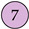 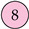
MinAV	∅	∅	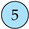 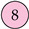

**Table 6:** Selected features by channels for RFE method.

Features	Channels for 75 Features	Channels for 65 Features
Kurtosis	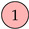	∅
Skewness	∅	∅
SSI	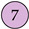	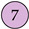
SD	∅	∅
RMS	∅	∅
Entropy	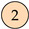 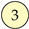 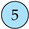 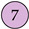	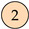 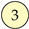 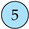 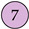
MAV	∅	∅
AAC	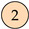 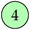 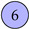 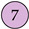	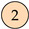 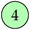 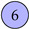 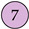
DASDV	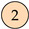	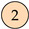
LD	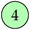 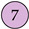	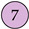
MMAV1	∅	∅
MMAV2	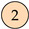 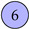	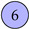
SSC	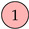 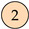 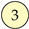 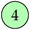 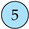 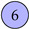 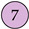 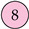	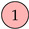 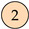 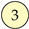 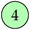 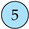 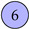 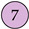 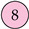
WAMP	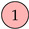 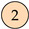 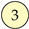 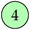 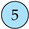 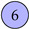 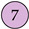 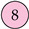	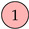 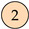 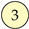 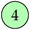 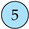 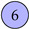 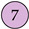 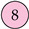
WL	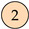 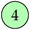 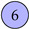 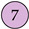	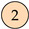 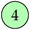 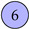 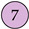
VAR	∅	∅
RSSQ	∅	∅
MNF	∅	∅
MDF	∅	∅
PKF	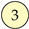 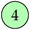	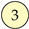 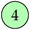
P2P	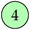	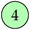
BP	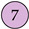	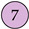
TM(3)	∅	∅
TM(5)	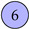	∅
V0	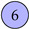	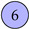
MAD0	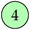	∅
MAD1	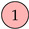 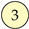 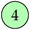 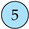	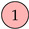 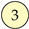 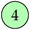
IEMG	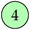	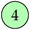
MaxAV	∅	∅
ZC	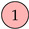 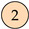 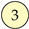 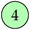 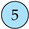 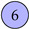 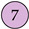 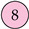	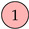 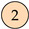 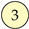 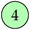 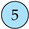 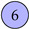 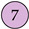 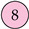
AVSER	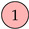 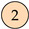 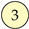 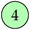 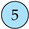 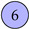 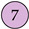	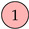 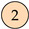 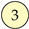 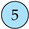 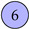 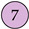
AVSSR	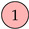 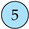 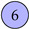 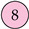	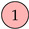 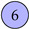 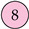
COV	∅	∅
DAMV	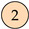 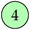 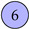 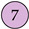	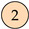 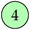 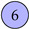 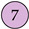
IQR	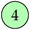	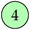
MVSR	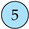 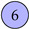 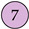 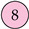	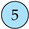 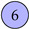
MinAV	∅	∅

## Data Availability

Data are contained within the article.
